# Reallocating bouted sedentary time to non-bouted sedentary time, light activity and moderate-vigorous physical activity in adults with prediabetes and type 2 diabetes

**DOI:** 10.1371/journal.pone.0181053

**Published:** 2017-07-28

**Authors:** Jenny Rossen, Matthew P. Buman, Unn-Britt Johansson, Agneta Yngve, Barbara Ainsworth, Kerstin Brismar, Maria Hagströmer

**Affiliations:** 1 Sophiahemmet University, Stockholm, Sweden; 2 Department of Clinical Science and Education, Södersjukhuset, Karolinska Institutet, Stockholm, Sweden; 3 Arizona State University, School of Nutrition and Health Promotion, College of Health Solutions, Phoenix, Arizona, United States of America; 4 Department of Food, Nutrition and Dietetics, Uppsala University, Uppsala, Sweden; 5 Department of Molecular Medicine and Surgery, Karolinska Institutet/ Rolf Luft Research Center for Diabetes and Endocrinology, Karolinska University Hospital, Stockholm, Sweden; 6 Department of Neurobiology, Care Sciences and Society, Division of Physiotherapy, Karolinska Institutet, Stockholm, Sweden; 7 Functional Area Occupational Therapy & Physiotherapy, Allied Health Professionals Function, Karolinska University Hospital, Stockholm, Sweden; Vanderbilt University, UNITED STATES

## Abstract

**Aim:**

The aim of this study was to investigate the potential associations of reallocating 30 minutes sedentary time in long bouts (>60 min) to sedentary time in non-bouts, light intensity physical activity (LPA) and moderate- to vigorous physical activity (MVPA) with cardiometabolic risk factors in a population diagnosed with prediabetes or type 2 diabetes.

**Methods:**

Participants diagnosed with prediabetes and type 2 diabetes (n = 124, 50% men, mean [SD] age = 63.8 [7.5] years) were recruited to the physical activity intervention Sophia Step Study. For this study baseline data was used with a cross-sectional design. Time spent in sedentary behaviors in bouts (>60 min) and non-bouts (accrued in <60 min bouts) and physical activity was measured using the ActiGraph GT1M. Associations of reallocating bouted sedentary time to non-bouted sedentary time, LPA and MVPA with cardiometabolic risk factors were examined using an isotemporal substitution framework with linear regression models.

**Results:**

Reallocating 30 minutes sedentary time in bouts to MVPA was associated with lower waist circumference (b = -4.30 95% CI:-7.23, -1.38 cm), lower BMI (b = -1.46 95% CI:-2.60, -0.33 kg/m2) and higher HDL cholesterol levels (b = 0.11 95% CI: 0.02, 0.21 kg/m^2^. Similar associations were seen for reallocation of sedentary time in non-bouts to MVPA. Reallocating sedentary time in bouts to LPA was associated only with lower waist circumference.

**Conclusion:**

Reallocation of sedentary time in bouts as well as non-bouts to MVPA, but not to LPA, was beneficially associated with waist circumference, BMI and HDL cholesterol in individuals with prediabetes and type 2 diabetes. The results of this study confirm the importance of reallocation sedentary time to MVPA.

## Introduction

Individuals with prediabetes and type 2 diabetes are recommended to engage in at least 150 min/week of moderate-to vigorous intensity physical activity (MVPA) [1]. Moderate intensity physical activity corresponds to repeated aerobic movement of large muscle groups such as walking, cycling, jogging and swimming (≥3 Metabolic Equivalents) [1, 2]. These recommendations are based on rigorous evidence [1, 3]. Yet, only a small proportion of the population with type 2 diabetes meets this recommendation [4, 5]. Reducing time in sedentary behavior (SB) and increasing time in light intensity physical activity (LPA) (standing, stepping and slow walking ≥1.6–2.9 Metabolic Equivalents) during the day is suggested as a first part of a stepped approach in increasing physical activity [2, 6, 7]. Emerging evidence also points at recommending interrupting SB alongside with being physically active. This may be especially beneficial to people with impaired glucose tolerance [8–10]. To underpin such recommendations more evidence is needed in terms of how often breaks in SB should be accomplished [11] and what intensity should be promoted to substitute the sedentary time [11, 12].

Physical activity is a complex behavior. During a day individuals are engaged in a pattern of behaviors at different intensities while awake: sedentary behavior (sitting/lying with low energy expenditure), standing and moving (stepping, walking, and exercising) at various bout lengths and with interruptions of different time lengths. When associating physical activity and SB with health parameters the whole-day pattern should be considered [6]. As time is finite, spending more time in one behavior inevitably reduces time in another behavior. For example, spending less time in SB may be replaced by time in LPA or in MVPA. When exploring the effects of reducing SB it is imperative to adjust for the behavior that it is replaced with to separate the effects [6, 11]. Isotemporal substitution methods have been suggested to model associations of reallocating time in one behavior for time in another with health [6, 13]. The model considers the fact that time is fixed (isotemporal) as well as the simultaneous relationships between activity intensities. A number of studies have used the isotemporal substitution paradigm in cross-sectional studies in both healthy populations e.g., [14, 15], in a population with impaired glucose control e.g. [16] and in populations with type 2 diabetes e.g., [17–19]. Beneficial associations with cardiometabolic risk factors have been demonstrated both of reallocating SB to MVPA e.g., [14–17, 19, 20] and to LPA e.g., [14, 16–18, 20], yet the evidence for LPA is still equivocal.

Recent studies also have reported health benefits of reallocating bouted sedentary time (≥ 30 min sedentary) to non-bouted sedentary time (sporadic sedentary time < 30 min) in persons with diabetes but the findings are inconclusive [17, 18]. Most research on breaks in SB is using 30 minutes or less as a break-point for bouted SB. To our knowledge evidence is scarce for longer time intervals to break up a sedentary bout in the population with diabetes and prediabetes (impaired glucose control). This population is spending a large proportion of waking time sedentary and it is likely that this is in prolonged periods. More evidence is needed on the doses and patterns of SB, whether all duration of SB is similarly harmful [6, 11, 21] and on the effects of substituting sedentary time with LPA versus MVPA [21].

The aim of this study was to investigate the associations of reallocating sedentary time in bouts (>60 min) to sedentary time in non-bouts, LPA or MVPA with cardiometabolic risk factors in a population diagnosed with prediabetes or type 2 diabetes.

## Materials and methods

### Participants

This study reports baseline data obtained April 2013-November 2015 on a sample collected from the Sophia Step Study. A detailed description about the data collection can be found in the study protocol [22]. Patients at the health care centers of Sophiahemmet in Stockholm, Sweden diagnosed with prediabetes and type 2 diabetes meeting inclusion criteria were invited to participate. A total of 134 participants diagnosed with prediabetes or diabetes where recruited and measured by their diabetes specialist nurse. Participants gave full informed consent to participate in the study, and ethical approval was obtained from the Regional Ethical Review Board in Stockholm (Dnr.2012/1570-31/3).

*Inclusion criteria*: 40–80 years; ability to communicate in Swedish; having prediabetes (HbA_1c_ > 39-<47 mmol/mol and/or fasting glucose >5.6 mmol/l) or diagnosed with type 2 diabetes for a duration of ≥1 year. *Exclusion criteria*: myocardial infarction in the past 6 months, serum creatinine >140 mmol/l, diabetic foot ulcer or risk of ulcer (severe peripheral neuropathy), on insulin since the last 6 months, additional disease prohibiting physical activity, repeated hypoglycemia or severe hypoglycemia in the past 12 months, being classified in “very hard-intensity activity” category” according to the Stanford Brief Activity Survey [23] and having no access to internet.

### Objectively measured physical activity and sedentary time

Time spent in MVPA, LPA, total sedentary time and breaks in sedentary time were measured using the ActiGraph GT1M accelerometer (ActiGraph, Pensacola, FL). The participants were asked to wear the accelerometer placed on the lower back [24] during waking hours (except for swimming and bathing) for seven consecutive days. The accelerations were sampled at 10 Hz and data were summarized over one minute using ActiLife v.6.13.1 software. After comparison of wear time from participant’s logs, non-wear time was set at >90 minutes consecutive zero counts with allowance for two minutes interval of nonzero counts [25].

Data of more than 10 hours per day for more than 3 days were included in the analyses [26]. Valid wear time was divided into categories based on existing count-based thresholds SB <100 counts per minute (cpm) [27]; LPA (100–1,951 cpm) and MVPA (≥1,952 cpm) [28]. Sedentary time was further split into “bouted” sedentary time based on uninterrupted periods of sedentary time of >60 minutes and non-bouted, interrupted, sedentary time. Bouted sedentary time was defined as >60min bouts of <100 cpm with allowance for breaks ≥100 cpm <1 min and non-bouted sedentary time as all other sedentary time (< 100 cpm ≤59 minutes). If there was a break of ≥100 cpm >1 minute that time was considered either as LPA or MVPA. Sedentary time in non-bouts is sedentary time with breaks more often than after 60 minutes. Bouted sedentary time is hereafter referred to as SB60 bouts and interrupted sedentary time as non-bouted SB. For explorative purposes the data was also split into >40 and > 20 minutes sedentary bouts and non-bouts.

### Cardiometabolic biomarkers

Measurements included fasting blood samples of HbA_1c_ (mmol/mol and %), fasting plasma glucose (mmol/l), triglycerides (mmol/l), HDL cholesterol (mmol/l), LDL cholesterol (mmol/l) and resting systolic and diastolic blood pressure (mmHg). Analyzing methods for blood samples has been described in detail elsewhere [22]. Resting blood pressure was measured with Omron M6 Comfort. Anthropometric measurements included weight using a Tanita digital scale (Model TBF-300A, Arlington Heights, IL). Weight was measured with light clothing and no shoes to the nearest 0.1 kilogram. Height was measured at the first visit by use of a calibrated stadiometer to the nearest centimeter. Waist circumference was measured with SECA 201 tape, horizontal around the waist 2 cm above the umbilicus.

### Sociodemographic and health behavior/status covariates

Sociodemographic variables included age, gender and education (university studies or not). Health status and use of medication was drawn from the medical records. Current medication (insulin, anti-diabetic tablets, statins and other cardiovascular disease (CVD) medications) was dichotomized into use of diabetes medication or not and use of CVD medication or not. Having other diseases (hyperlipidemia, hypertension, other CVDs (coronary heart disease and peripheral vascular disease), cancer, chronic obstructive pulmonary disease, inflammatory disease and other diseases) was dichotomized into having more than two other diseases or not. A questionnaire was e-mailed to the participants exploring dietary habits (type of cooking fat, amount fiber rich bread slices weekly) and daily fruits and vegetable intake) [29] and sleep quality (estimation of overall sleep quality, rating scale 1–5).

### Statistical analysis

All outcome variables were examined for normality and natural log transformations were performed for HbA1c, plasma glucose, triglycerides, systolic and diastolic blood pressure. For waist circumference, BMI, HDL cholesterol and LDL cholesterol, data approximated a normal distribution, the original values were retained and results are presented as unstandardized regression coefficients. For HbA1c, plasma glucose, triglycerides, systolic and diastolic blood pressure the unstandardized regression coefficients were back-transformed and are presented as relative rates (changes in the natural log of a variable are interpretable as percentage changes).

Covariate analyses were performed for each independent outcome. Age was included as a covariate in all models. All other covariates were retained only if p-value was < 0.2 when all covariates were included in the model. Final retained covariates in each model are described in S1 Table.

Isotemporal substitution analysis was conducted to study the potential association of reallocating bouted sedentary time to different activity intensity and non-bouted sedentary time [13]. Regression models were used to explore associations of the exposure variables (bouted and non-bouted SB, LPA and MVPA) with cardiometabolic risk factors. All exposure variables were first converted to 30 min/day units to aid in interpretation of the regression coefficients. First, a single regression model examined the raw associations of each exposure and the health outcome with only covariates and wear time in the model. Second, a partition model examined the associations of each exposure variable after adjusting for all other exposure variables and covariates. Finally, isotemporal substitution models the associations of reallocating one exposure variable for another (e.g., SB60 bouts to MVPA), while adjusting for covariates as well as time in other activities. This was accomplished by including a total time variable (wear time based on Choi et al [25] in the model and dropping the exposure variable of interest. By replacing 30 minutes in one activity (e.g., SB60 bouts) with total time, the coefficient can be interpreted as the effect of replacing 30 minutes of SB60 bouts with the respective other activity in the regression (e.g., 30 minutes of MVPA). Additional analyses with sedentary time in sets of >40- and >20- minutes sedentary bouts and non-bouts, respectively, was also conducted for explorative purposes and are reported as supplementary material. Differences of interest were >1.5 kg/m^2^ BMI, >1.13 mmol/l HDL cholesterol [30] and >4 mmol/l HbA1c [31].

IBM SPSS version 23.0 was used for all statistical analyses.

## Results

From 134 enrolled participants, 124 (93%) had valid accelerometer data and cardiometabolic biomarkers for inclusion in analyses. Mean age was 63.8 ± 7.5 years, 62 were women (50%), mean BMI was 29.7 ± 4.6. kg/m^2^ and a large proportion (60%) had university education. Table 1 displays participants characteristics per diagnose prediabetes or type 2 diabetes. Participants with type 2 diabetes had significantly higher (p< 0.05) levels of HbA1c, plasma glucose, LDL cholesterol and triglycerides and a higher proportion had other diseases compared to participants with prediabetes. Participants with prediabetes spent significantly more total time in MVPA and a higher proportion had a university education. The mean duration of type 2 diabetes was 9.6 years. Most of the participants with type 2 diabetes (82.2%) and none of the participants with prediabetes used antidiabetic medication. A majority (79% diabetes, 64% prediabetes) used CVD medication.

**Table 1:** Participant demographics by prediabetes and type 2 diabetes status.

n	Prediabetes 33	Type 2 diabetes 91	p	Total 124
**Age, years**	64.8 (7.4)	63.5 (7.5)	0.375	63.8 (7.5)
**Female**	23	39		62 (50)
**University degree, yes**	20 (66.7)	51 (57.3)	0.370	71 (59.7)
**Sleep quality, score**	2.3 (0.2)	2.4 (0.1)	0.592	2.26 (1.1)
**Medications**				
Insulin, yes	0	16 (17.8)	<0.09	16 (13.0)
Diabetes tablets, yes	0	67 (74.4)	<0.00	67 (54.5)
Statins, yes	12 (36.4)	54 (60.0)	0.020	66 (53.7)
Other CVD medication, yes	16 (48.5)	56 (62.2)	0.173	72 (58.5)
**Comorbidities**				
Hypertension, yes	23 (71.9)	66 (73.3)	0.875	89 (73.0)
Hyperlipidemia, yes	22 (68.8)	70 (77.8)	0.312	92 (75.4)
Other CVD^a^, yes	3 (9.4)	22 (24.7)	0.067	25 (20.7
Chronic obstructive pulmonary disease, yes	3 (9.4)	15 (16.7)	0.322	18 (14.8)
Inflammatory diseases, yes	2 (6.3)	3 (3.4)	0.487	5 (4.1)
Cancer, yes	2 (6.3)	7 (7.9)	0.768	9 (7.4)
Other disease, yes	9 (29.0)	21 (23.6)	0.551	30 (25.0)
**Diet quality**				
Fruits and vegetables, daily servings	2.9 (2.2)	2.8 (2.2)	0.862	2.8 (2.2)
Cooking fat, butter^b^	1,6 (0.5)	1.7 (0.5)	0.421	1.7 (0.5)
Fiber from bread, weekly slices	14.0 (10.3)	15.3 (11.3)	0.515	14.9 (11.0)
**Cardio-metabolic biomarkers**				
Body Mass Index, kg/m^2^	29.0 (3.5)	30.0 (5.0)	0.314	29.7 (4.6)
Waist circumference men, cm	105.0 (13.3)	108.5 (10.8)	0.367	108.0 (11.2)
Waist circumference women, cm	96.8 (10.7)	100.5 (14.0)	0.281	99.2 (12.9)
Systolic blood pressure, mmHg	126.7 (16.7)	132.0 (14.3)	0.088	130.6 (15.1)
Diastolic blood pressure, mmHg	81.3 (9.3)	83.7 (9.2)	0.210	83.1 (9.3)
HbA_1c_, mmol/mol (IFCC)	39.8 (4.1)	51.1 (11.0)	<0.000	48.1 (10.8)
HbA_1c_, % (DCCT)	5.7 (0.4)	6.8 (1.1)	<0.000	6.5 (1.0)
Fasting plasma glucose, mmol/L	6.3 (0.7)	7.8 (1.7)	<0.000	7.4 (1.6)
C-Peptide nmol/L	1.8 (1.4)	1.1 (0.5)	0.683	1.1 (0.8)
HDL cholesterol, mmol/L	1.6 (0.4)	1.4 (0.4)	0.007	1.4 (0.4)
LDL cholesterol, mmol/L	3.4 (0.7)	2.8 (1.0)	0.001	3.0 (1.0)
Triglycerides, mmol/L	1.4 (0.5)	1.8 (0.9)	0.032	1.7 (0.9)
**Physical activity and sedentary behavior**				
Accelerometer wear time, min/day	836,8 (79.9)	835.7 (82.3)	0.833	834 (80.3)
Moderate to vigorous intensity physical activity (MVPA), min/day	44.7 (28.4)	30.5 (21.0)	0.003	34.1 (24.1)
Light physical activity (LPA), min/day	242.4 (60.9)	222.7 (64.4)	0.134	224.4 (63.1)
Total sedentary time, min/day	549.7 (93.7)	582.8 (78.5)	0.067	576.1 (82.0)
Time in sedentary bouts >60 min, min/day	96.4 (79.5)	118.7 (71.8)	0.161	113.6 (73.5)
Number of sedentary bouts >60 min, n	1.2 (0.9)	1.5 (0.9)	0.097	1.4 (0.9)

Table presents mean (standard deviation), sample n (%) or amount.

^a^ Coronary heart disease and peripheral vascular disease

^b^ Fat quality was dichotomized into Butter or oils and margarine

The raw associations of each exposure variable, adjusted for wear time, age and covariates, with cardiometabolic biomarkers (single model), are shown in S2 Table.

Results for biomarkers with significant associations (p<0.05) in the partition model are displayed in Table 2. When each exposure variable was adjusted also for time in other activities, beneficial associations for MVPA remained significant with BMI, waist circumference and HDL cholesterol. S3 Table shows the results of the partition model for total sedentary time, sedentary bouts of >60/40/20 minutes and non-bouts.

**Table 2:** Partition model for associations of each activity with cardio-metabolic biomarkers, adjusted for time in the other activities.

Regression coefficients (95% CI)				Relative rate (95% CI)	
	Waist circumference (cm)	BMI (kg/m^2^)	HDL cholesterol (mmol/l)	HbA1c (mmol/mol)	Fasting plasma glucose (mmol/mol)
SB60 bouts	0.94 (-0.22, 2.10)	0.16 (-0.29, 0.61)	0.00 (-0.04, 0.04)	**1.02 (1.00, 1.04)**	1.00 (0.98, 1.02)
Non-bouted SB	0.32 (-0.67, 1.31)	-0.02 (-0.40, 0.36)	-0.02 (-0.06, 0.01)	-0.02 (-0.06, 0.01)	**0.98 (0.97, 1.00)**
LPA	-0.21 (-1.47, 1.05)	-0.12 (-0.60, 0.37)	0.01 (-0.03, 0.05)	1.01 (0.99, 1.03)	1.01 (0.98, 1.03)
MVPA	**-3.36 (-6.14, -0.59)**	**-1.31 (-2.38, -0.24)**	**0.12 (0.03, 0.21)**	1.02 (0.97, 1.07)	1.02 (0.96, 1.07)

Sedentary time was divided in 60 minutes or longer bouts (SB60 bouts) and other sedentary time (Non-bouted SB).

Bold indicates significant results (p<0.05)

Physical activity intensity thresholds are <100 counts/min for sedentary (SB), 100 to 1951 counts/min for light intensity physical activity (LPA) and ≥1952 for moderate and vigorous physical activity (MVPA).

Table 3 shows significant results (p<0.05) from the isotemporal substitution analyses conducted for each exposure variable. Beneficial associations of similar effect size were observed for waist circumference, BMI and HDL cholesterol when 30 minutes of both SB60 bouts and non-bouted SB was reallocated to MVPA. Reallocating 30 minutes of SB60 bouts to LPA was also beneficially associated with waist circumference.

**Table 3:** Isotemporal substitution model for associations of reallocated time in one activity to another activity with cardio-metabolic biomarkers.

Reallocation of:	Regression coefficients (95% CI)		
	Waist circumference (cm)	BMI (kg/m^2^)	HDL cholesterol (mmol/l)
SB60 bouts to non-bouted SB^a^	-0.62 (-1.78, 0.53)	-0.18 (-0.62, 0.27)	-0.03 (-0.07, 0.01)
SB60 bouts to LPA	**-1.15 (-2.27, -0.03)**	-0.27 (-0.70, 0.15)	0.01 (-0.03, 0.05)
SB60 bouts to MVPA	**-4.30 (-7.23, -1.38)**	**-1.46 (-2.60, -0.33)**	**0.11 (0.02, 0.21)**
Non-bouted SB to LPA	-0.53 (-1.95, 0.89)	-0.10 (-0.65, 0.45)	0.04 (-0.01, 0.08)
Non-bouted SB to MVPA	**-3.68 (-6.54, -0.83)**	**-1.29 (-2.39, -0.19)**	**0.14 (0.05, 0.23)**
LPA to MVPA	-3.15 (-6.38, 0.08)	-1.19 (-2.43, 0.05)	0.11 (-0.00, 0,21)

Bold indicates significant results (p<0.05).

Physical activity intensity thresholds are <100 counts/min for sedentary (SB), 100 to 1951 counts/min for light intensity physical activity (LPA) and ≥1952 for moderate and vigorous physical activity (MVPA).

Total time in each intensity was divided by a constant of 30, so that one unit of increase corresponds to 30 min exchange between one activity to another.

Total wear time was then entered together with covariates and all activities but one, simulating reallocation of 30 min in one activity by 30 min in another activity. By dropping each activity one by one the reallocating of time from different activities is modelled.

^a^ Sedentary time was divided in 60 minutes or longer bouts (SB60 bouts) and other sedentary time (Non-bouted SB60).

The same associations were observed when sedentary time was bouted in >40- and >20 minutes bouts, with the addition that reallocating 30 min from LPA to MVPA was beneficially associated with waist circumference, BMI and HDL cholesterol (S4 Table).

Not shown in Table 3, due to very small beta coefficients, are the associations of SB60 bouts to LPA with diastolic blood pressure (RR = 0.99).

## Discussion

The findings of this study add to previous findings [17] that reallocating objectively measured time from SB to MVPA is beneficially associated with BMI, waist circumference and HDL cholesterol in persons with prediabetes and type 2 diabetes. The associations were of similar effect size when reallocating both bouted and non-bouted sedentary time to MVPA. Previous studies using the isotemporal method have reported significant beneficial associations of reallocating bouted SB (> 30 min) also to LPA with waist circumference and BMI on similar populations [17, 18] and of reallocating 30 min of total SB to LPA with cardiometabolic benefits on healthy populations [14, 15]. In this study, the only association of reallocating SB to LPA was a slightly lower waist circumference when 30 minutes of bouted sedentary time (>60 min) was reallocated to LPA. Also in contrast to what has been reported earlier [17, 18], although there were small associations, we did not find significant associations when reallocating SB60 bouts to non-bouted SB.

When splitting sedentary behavior in to >40- and >20 minutes sedentary bouts and non-bouts we found, consistent with previous findings, that reallocating time from LPA to MVPA was beneficially associated with BMI, waist circumference [17] and HDL cholesterol [14]. This implies that 30 minutes increase in MVPA has the most potential benefits on obesity parameters and HDL cholesterol. Several authors have demonstrated considerably stronger health associations by reallocating SB to MVPA than to LPA [11, 14–16, 19, 20].

The non-significant associations of reallocating SB60 bouts to non-bouted SB and SB to LPA in this study cannot be ruled out, but can be explained by the low amount of time spent in sedentary bouts (>60 min) and the possible dose-response relationship of physical activity and health. More time is probably needed to be reallocated from SB to LPA and for bouted to non-bouts for potential associations. A recommendation to decrease total SB with 6o min/day has been proposed [6, 11] and if we would have chosen 60 min/day as units to be reallocated, the effect size would have been doubled. More research is needed on the health benefits of different patterns of activities and sedentary behavior, e.g. stratifying for number of sedentary bouts of different lengths and time in MVPA as well as different subgroups of the population with impaired glucose metabolism.

The inconsistency between this study and other studies on populations with type 2 diabetes can be explained by differences in terms of sample size, age range, gender distribution, disease duration and the fact that this study included persons with both prediabetes and type 2 diabetes. In this study the population had quite high education level, was more active than the general Swedish population with type 2 diabetes and the diabetes seemed to be well managed (the group with type 2 diabetes meet the target level for many clinical variables). Furthermore, using different devices for measuring physical activity and sedentary behavior and differences in the algorithms used to classify the data should be considered when making comparisons.

The three models revealed very small and mixed results on the metabolic parameters HbA1c and plasma glucose. None of the associations found in the single and partition model remained in the isotemporal model. Type 2 diabetes is a progressive condition and the included participants had various durations of both prediabetes and type 2 diabetes. The average HbA1c for the group with type 2 diabetes was below the Swedish target levels for populations with type 2 diabetes [32]. We did not control for the dose or type of medication and the absence of positive associations can be due to the fact that this was a well-medicated group of participants with type 2 diabetes and the inclusion of individuals with prediabetes. A broad spectrum of individuals across the diabetes continuum, including those at risk for diabetes as well as those with type 2 diabetes as well a wide age range was included in this study. Although this makes the interpretation of the results more difficult, it increases generalizability of our results and contributes to the knowledge of reducing risk and severity for type 2 diabetes.

In this study, reallocation of 30 minutes sedentary time to MVPA during a day was associated with clinically important differences of 1.46 kg/m^2^ in BMI and 0.14 mmol/l in HDLcholesterol. A reduction of 1.46 kg/m^2^ in BMI implies ~4 kg for individuals of 171 cm height and a weight difference of 5%, which has been confirmed to reduce the cardiovascular risk [30]. A difference of 0.14 mmol/l in HDL cholesterol can potentially lower the risk of coronary heart disease [33]. However, the results were not fully powered (0.71 and 0.79 for BMI and HDL cholesterol respectively) and the findings need to be confirmed by other data sets to eliminate the risk of false positive findings.

Strengths of this study include the objective measurements of physical activity and time spent sedentary and the range of outcome variables measured in a standardized way. Also, diet and sleep, factors influencing the outcomes were controlled for in the analyses.

Objective measures such as accelerometry are shown to have higher accuracy compared to self-reported levels of SB, LPA and MVPA. Yet, objective measures have some limitations. When exploring sedentary time, the data reduction procedure used to separate non-wear time from zero counts is a critical step and accelerometers are not sensitive enough to exclude misclassification of actual wear time as sedentary time and vice versa. In this study we chose >90 minutes of consecutive zero counts (with allowance for two minutes interval of nonzero counts min) as non-wear time. This allowed for a daytime nap to be included as wear time and reduced the risk of excluding actual wear time. Moreover, by using accelerometers placed on the lower back we cannot separate sitting from standing. As a breakpoint for non-sedentary activity we have used > 1 min breaks with cpm ≥ 100. Hence, this is regarded as LPA, corresponding to stepping or moving slowly for at least one minute and should not be mixed up with standing.

The pattern of sporadic, bouted and embedded MVPA and LPA was not considered in this study. We have used total time spent in LPA and MVPA, including all time >1 min and did not divide these activity intensities into bouts, thus 30 min MVPA could be accumulated during the day and every minute had potential health enhancing effects. Time in MVPA is often embedded in LPA [34] and shorter sedentary bouts and interrupted sedentary time are possibly correlated with LPA. We did not adjust for the number of breaks of 1 minute or less during the bouted sedentary time, which can have health enhancing effects. The cut points used for MVPA and LPA should be considered with some caution as they are absolute and do not consider the fact that the potential health effects contributed by MVPA and LPA depends on current fitness level, age, genetic dispositions and other circumstances. In future studies, we suggest to control for fitness levels, as the most unfit individuals seem to benefit also at lower activity intensities [20].

The study has some limitations and interpretation of the associations must be made with caution. The participants had volunteered for a physical activity intervention and more than half were under both CVD and diabetes treatment. The data are cross-sectional and does not imply a causal relationship, yet the isotemporal models simulate changes, which can be considered as strength. The multiple regression analyses were run without adjusting the alpha level of significance and hence false positive errors cannot be ruled out. However, the number of statistical analyses run where reduced by including only biomarkers with significant results (p<0.05) from the single to the partition analysis and from the partition to the isotemporal analysis.

Reducing time spent sedentary and frequently breaking up sitting could be parts of a stepped approach for better health, but emphasis should be to collect minutes of MVPA during the day. Advice must be individualized depending on the current individual physical activity pattern, fitness level, possibilities, readiness, daily routines and preferences.

## Conclusion

Reallocation of sedentary time in bouts as well as non-bouts to MVPA, but not to LPA, is beneficially associated with waist circumference, BMI and HDL cholesterol in individuals with prediabetes and type 2 diabetes. The results of this study confirm the importance of reallocation sedentary time to MVPA.

## Supporting information

S1 Table Co-variates showing P<0.2 in co-variate analysis were remained in the following analyses. Age was adjusted for in all further analyses. Initially considered co-variates in all co-variates analyses were: gender; education (university studies or not), use of diabetes medication (insulin and oral tablets) or not; use of CVD medication or not; having more than to other diseases (including lung disease, hypertension, hyperlipidemia, other CVD (coronary heart disease and peripheral vascular disease), inflammatory disease, other co-morbidity and cancer) or not; sleep quality; fiber from bread (number slices per week), amount servings of fruits and vegetable per day and cooking fat quality.
(PDF)

S2 Table Single model for cross-sectional associations of each 30 minutes per day of long bout sedentary time, non-bouted sedentary time, light intensity physical activity (LPA) and moderate to vigorous intensity (MVPA) with cardio-metabolic biomarkers. Bold indicates significant results (p<0.05)
Activity intensity tresholds are <100 counts/min for sedentary (SB), 100 to 1951 counts/min for LPA and ≥1952 for MVPA.
^a^ Sedentary time was divided in 60 minutes or longer bouts (SB60) and non-bouted sedentary time (Non-bouted SB60).
^b^ Sedentary time was divided in 40 minutes or longer bouts (SB40) and non-bouted sedentary time (Non-bouted SB40).
^c^ Sedentary time was divided in 20 minutes or longer bouts (SB20) and non-bouted sedentary time (Non-bouted SB).</SI_Caption>
(PDF)

S3 Table Partition model for cross-sectional associations of each 30 minutes per day of long bout sedentary time, non-bouted sedentary time, light intensity physical activity (LPA) and moderate to vigorous intensity (MVPA) with cardio-metabolic biomarkers, adjusted for time in the other activities. Bold indicates significant results (p<0.05)
Activity intensity tresholds are <100 counts/min for sedentary (SB), 100 to 1951 counts/min for light intensity physical activity (LPA) and ≥1952 for MVPA.
^a^ Sedentary time was divided in 60 minutes or longer bouts (SB60) and non-bouted sedentary time (Non-bouted SB60).
^b^ Sedentary time was divided in 40 minutes or longer bouts (SB40) and non-bouted sedentary time (Non-bouted SB40).
^c^ Sedentary time was divided in 20 minutes or longer bouts (SB20) and non-bouted sedentary time (Non-bouted SB20).</SI_Caption>
(PDF)

S4 Table Isotemporal substitution model for associations of reallocated time in one activity to another activity with cardio-metabolic biomarkers. Bold indicates significant results (p<0.05).
Activity intensity thresholds are <100 counts/min for sedentary (SB), 100 to 1951 counts/min for light intensity physical activity and ≥1952 for MVPA.
Total time in each intensity was divided by a constant of 30, so that one unit of increase corresponds to 30 min exchange between one activity to another.
Total wear time was then entered together with covariates and all activities but one, simulating reallocation of 30 min in one activity by 30 min in another activity. By dropping each activity one by one the reallocating of time from different activities is modelled.
^a^ Sedentary time was divided in 60 minutes or longer bouts (SB60) and other sedentary time (Non-bouted SB60).
^b^ Sedentary time was divided in 40 minutes or longer bouts (SB40) and other sedentary time (Non-bouted SB40).
^c^ Sedentary time was divided in 20 minutes or longer bouts (SB20) and other sedentary time (Non-bouted SB20).</SI_Caption>
(PDF)

## References

[1] Colberg SR, Sigal RJ, Yardley JE, Riddell MC, Dunstan DW, Dempsey PC, et al Physical Activity/Exercise and Diabetes: A Position Statement of the American Diabetes Association. Diabetes Care. 2016;39(11):2065–79. doi: 10.2337/dc16-1728 27926890 10.2337/dc16-1728 PMC6908414

[2] Ainsworth BE, Haskell WL, Leon AS, Jacobs DR Jr., Montoye HJ, Sallis JF, et al Compendium of physical activities: classification of energy costs of human physical activities. Med Sci Sports Exerc. 1993 1;25(1):71–80. . Epub 1993/01/01. Eng. 8292105 10.1249/00005768-199301000-00011

[3] Colberg SR, Albright AL, Blissmer BJ, Braun B, Chasan-Taber L, Fernhall B, et al Exercise and type 2 diabetes: American College of Sports Medicine and the American Diabetes Association: joint position statement. Exercise and type 2 diabetes. Med Sci Sports Exerc. 2010 12;42(12):2282–303. doi: 10.1249/MSS.0b013e3181eeb61c . 21084931 10.1249/MSS.0b013e3181eeb61c

[4] Zethelius B, Gudbjornsdottir S, Eliasson B, Eeg-Olofsson K, Cederholm J, Swedish National Diabetes R. Level of physical activity associated with risk of cardiovascular diseases and mortality in patients with type-2 diabetes: report from the Swedish National Diabetes Register. Eur J Prev Cardiol. 2014 2;21(2):244–51. doi: 10.1177/2047487313510893 . 24227183 10.1177/2047487313510893

[5] Unick JL, Gaussoin SA, Hill JO, Jakicic JM, Bond DS, Hellgren M, et al Four-Year Physical Activity Levels among Intervention Participants with Type 2 Diabetes. Medicine and science in sports and exercise. 2016 12;48(12):2437–45. doi: 10.1249/MSS.0000000000001054 . Epub 2016/07/30. eng. 27471785 10.1249/MSS.0000000000001054 PMC5110392

[6] Dempsey PC, Owen N, Yates TE, Kingwell BA, Dunstan DW. Sitting Less and Moving More: Improved Glycaemic Control for Type 2 Diabetes Prevention and Management. Curr Diab Rep. 2016 11;16(11):114 doi: 10.1007/s11892-016-0797-4 . Epub 2016/10/05. eng. 27699700 10.1007/s11892-016-0797-4

[7] Henson J, Dunstan DW, Davies MJ, Yates T. Sedentary behaviour as a new behavioural target in the prevention and treatment of type 2 diabetes. Diabetes Metab Res Rev. 2016 1;32 Suppl 1:213–20. doi: 10.1002/dmrr.2759 . Epub 2016/01/28. Eng. 26813615 10.1002/dmrr.2759

[8] Dunstan DW, Kingwell BA, Larsen R, Healy GN, Cerin E, Hamilton MT, et al Breaking up prolonged sitting reduces postprandial glucose and insulin responses. Diabetes Care. 2012 5;35(5):976–83. doi: 10.2337/dc11-1931 . Epub 2012/03/01. Eng. 22374636 10.2337/dc11-1931 PMC3329818

[9] Henson J, Davies MJ, Bodicoat DH, Edwardson CL, Gill JM, Stensel DJ, et al Breaking Up Prolonged Sitting With Standing or Walking Attenuates the Postprandial Metabolic Response in Postmenopausal Women: A Randomized Acute Study. Diabetes Care. 2016 1;39(1):130–8. doi: 10.2337/dc15-1240 . 26628415 10.2337/dc15-1240

[10] Cooper AR, Sebire S, Montgomery AA, Peters TJ, Sharp DJ, Jackson N, et al Sedentary time, breaks in sedentary time and metabolic variables in people with newly diagnosed type 2 diabetes. Diabetologia. 2012 3;55(3):589–99. doi: 10.1007/s00125-011-2408-x . 22167127 10.1007/s00125-011-2408-x

[11] Matthews CE, Moore SC, Sampson J, Blair A, Xiao Q, Keadle SK, et al Mortality Benefits for Replacing Sitting Time with Different Physical Activities. Med Sci Sports Exerc. 2015 9;47(9):1833–40. doi: 10.1249/MSS.0000000000000621 . 25628179 10.1249/MSS.0000000000000621 PMC4515413

[12] Ekelund U, Steene-Johannessen J, Brown WJ, Fagerland MW, Owen N, Powell KE, et al Does physical activity attenuate, or even eliminate, the detrimental association of sitting time with mortality? A harmonised meta-analysis of data from more than 1 million men and women. Lancet. 2016 7 27 doi: 10.1016/S0140-6736(16)30370-1 . 27475271 10.1016/S0140-6736(16)30370-1

[13] Mekary RA, Feskanich D, Malspeis S, Hu FB, Willett WC, Field AE. Physical activity patterns and prevention of weight gain in premenopausal women. Int J Obes (Lond). 2009 9;33(9):1039–47. doi: 10.1038/ijo.2009.127 . 19546868 10.1038/ijo.2009.127 PMC2746452

[14] Buman MP, Winkler EA, Kurka JM, Hekler EB, Baldwin CM, Owen N, et al Reallocating time to sleep, sedentary behaviors, or active behaviors: associations with cardiovascular disease risk biomarkers, NHANES 2005–2006. Am J Epidemiol. 2014 2 1;179(3):323–34. doi: 10.1093/aje/kwt292 . 24318278 10.1093/aje/kwt292

[15] Ekblom-Bak E, Ekblom O, Bergstrom G, Borjesson M. Isotemporal substitution of sedentary time by physical activity of different intensities and bout lengths, and its associations with metabolic risk. Eur J Prev Cardiol. 2016 6;23(9):967–74. doi: 10.1177/2047487315619734 . 26635358 10.1177/2047487315619734

[16] Yates T, Henson J, Edwardson C, Dunstan D, Bodicoat DH, Khunti K, et al Objectively measured sedentary time and associations with insulin sensitivity: Importance of reallocating sedentary time to physical activity. Prev Med. 2015 7;76:79–83. doi: 10.1016/j.ypmed.2015.04.005 . 25900801 10.1016/j.ypmed.2015.04.005

[17] Falconer CL, Page AS, Andrews RC, Cooper AR. The Potential Impact of Displacing Sedentary Time in Adults with Type 2 Diabetes. Med Sci Sports Exerc. 2015 10;47(10):2070–5. doi: 10.1249/MSS.0000000000000651 . 26378943 10.1249/MSS.0000000000000651 PMC5131683

[18] Healy GN, Winkler EA, Brakenridge CL, Reeves MM, Eakin EG. Accelerometer-derived sedentary and physical activity time in overweight/obese adults with type 2 diabetes: cross-sectional associations with cardiometabolic biomarkers. PLoS One. 2015;10(3):e0119140 doi: 10.1371/journal.pone.0119140 . 25775249 10.1371/journal.pone.0119140 PMC4361561

[19] Hamer M, Hackett RA, Bostock S, Lazzarino AI, Carvalho LA, Steptoe A. Objectively assessed physical activity, adiposity, and inflammatory markers in people with type 2 diabetes. BMJ Open Diabetes Res Care. 2014;2(1):e000030 doi: 10.1136/bmjdrc-2014-000030 . 25452870 10.1136/bmjdrc-2014-000030 PMC4212571

[20] Ekblom-Bak E, Ekblom O, Bolam KA, Ekblom B, Bergstrom G, Borjesson M. SCAPIS Pilot Study: Sitness, Fitness and Fatness—Is Sedentary Time Substitution by Physical Activity Equally Important for Everyone's Markers of Glucose Regulation? Journal of physical activity & health. 2016 7;13(7):697–703. doi: 10.1123/jpah.2015-0611 . 26900674 10.1123/jpah.2015-0611

[21] Chastin SF, Egerton T, Leask C, Stamatakis E. Meta-analysis of the relationship between breaks in sedentary behavior and cardiometabolic health. Obesity (Silver Spring). 2015 9;23(9):1800–10. doi: 10.1002/oby.21180 . Epub 2015/08/27. eng. 26308477 10.1002/oby.21180

[22] Rossen J, Yngve A, Hagstromer M, Brismar K, Ainsworth BE, Iskull C, et al Physical activity promotion in the primary care setting in pre- and type 2 diabetes—the Sophia step study, an RCT. BMC Public Health. 2015;15:647 doi: 10.1186/s12889-015-1941-9 . 26164092 10.1186/s12889-015-1941-9 PMC4499440

[23] Taylor-Piliae RE, Norton LC, Haskell WL, Mahbouda MH, Fair JM, Iribarren C, et al Validation of a new brief physical activity survey among men and women aged 60–69 years. Am J Epidemiol. 2006 9 15;164(6):598–606. doi: 10.1093/aje/kwj248 . 16840522 10.1093/aje/kwj248

[24] Yngve A, Nilsson A, Sjostrom M, Ekelund U. Effect of monitor placement and of activity setting on the MTI accelerometer output. Medicine and science in sports and exercise. 2003 2;35(2):320–6. doi: 10.1249/01.MSS.0000048829.75758.A0 . Epub 2003/02/06. eng. 12569223 10.1249/01.MSS.0000048829.75758.A0

[25] Choi L, Liu Z, Matthews CE, Buchowski MS. Validation of accelerometer wear and nonwear time classification algorithm. Med Sci Sports Exerc. 2011 2;43(2):357–64. doi: 10.1249/MSS.0b013e3181ed61a3 . 20581716 10.1249/MSS.0b013e3181ed61a3 PMC3184184

[26] Hart TL, Swartz AM, Cashin SE, Strath SJ. How many days of monitoring predict physical activity and sedentary behaviour in older adults? Int J Behav Nutr Phys Act. 2011;8:62 doi: 10.1186/1479-5868-8-62 . 21679426 10.1186/1479-5868-8-62 PMC3130631

[27] Matthews CE, Chen KY, Freedson PS, Buchowski MS, Beech BM, Pate RR, et al Amount of time spent in sedentary behaviors in the United States, 2003–2004. Am J Epidemiol. 2008 4 1;167(7):875–81. doi: 10.1093/aje/kwm390 . Epub 2008/02/28. eng. 18303006 10.1093/aje/kwm390 PMC3527832

[28] Freedson PS, Melanson E, Sirard J. Calibration of the Computer Science and Applications, Inc. accelerometer. Med Sci Sports Exerc. 1998 5;30(5):777–81. . 9588623 10.1097/00005768-199805000-00021

[29] Sepp HB, U. Enkätfrågor om kost och fysisk aktivitet bland vuxna- Underlag till frågor i befolkningsinriktade enkäter Livsmedelsverket, 2004.

[30] Wing RR, Lang W, Wadden TA, Safford M, Knowler WC, Bertoni AG, et al Benefits of modest weight loss in improving cardiovascular risk factors in overweight and obese individuals with type 2 diabetes. Diabetes Care. 2011 7;34(7):1481–6. doi: 10.2337/dc10-2415 . Epub 2011/05/20. eng. 21593294 10.2337/dc10-2415 PMC3120182

[31] Bravata DM, Smith-Spangler C, Sundaram V, Gienger AL, Lin N, Lewis R, et al Using pedometers to increase physical activity and improve health: a systematic review. JAMA. 2007 11 21;298(19):2296–304. doi: 10.1001/jama.298.19.2296 . 18029834 10.1001/jama.298.19.2296

[32] Strath SJ, Kaminsky LA, Ainsworth BE, Ekelund U, Freedson PS, Gary RA, et al Guide to the assessment of physical activity: Clinical and research applications: a scientific statement from the American Heart Association. Circulation. 2013 11 12;128(20):2259–79. doi: 10.1161/01.cir.0000435708.67487.da . Epub 2013/10/16. Eng. 24126387 10.1161/01.cir.0000435708.67487.da

[33] Zethelius B, Eliasson B, Eeg-Olofsson K, Svensson AM, Gudbjornsdottir S, Cederholm J, et al A new model for 5-year risk of cardiovascular disease in type 2 diabetes, from the Swedish National Diabetes Register (NDR). Diabetes Res Clin Pract. 2011 8;93(2):276–84. doi: 10.1016/j.diabres.2011.05.037 . 21719139 10.1016/j.diabres.2011.05.037

[34] Robson J, Janssen I. A description of the volume and intensity of sporadic physical activity among adults. BMC Sports Sci Med Rehabil. 2015;7:2 doi: 10.1186/2052-1847-7-2 . Epub 2015/05/15. eng. 25973206 10.1186/2052-1847-7-2 PMC4429321

