# Supplementary material for: Reallocating bouted sedentary time to non-bouted sedentary time, light activity and moderate-vigorous physical activity in adults with prediabetes and type 2 diabetes
Source: PLoS One. 2017 Jul 28;12(7):e0181053. doi: 10.1371/journal.pone.0181053 (PMC5533318; doi:10.1371/journal.pone.0181053)
Supplement: S1 Table — Age was adjusted for in all further analyses. Initially considered co-variates in all co-variates analyses were: gender; education (university studies or not), use of diabetes medication (insulin and oral tablets) or not; use of CVD medication or not; having more than to other diseases (including lung disease, hypertension, hyperlipidemia, other CVD (coronary heart disease and peripheral vascular disease), inflammatory disease, other co-morbidity and cancer) or not; sleep quality; fiber from bread (number slices per week), amount servings of fruits and vegetable per day and cooking fat quality. (PDF) [file pone.0181053.s001.pdf]

**Outcome**	**Co-variates retained in the final analyses**
LDLcholesterol (mmol/l)	Diabetes medication, CVD medication, fiber intake
HDL cholesterol (mmol/l)	Gender, fruits and veg intake
TG (mmol/l)	Other diseases, fat quality, education
Fasting plasma glucose (mmol/l)	Diabetes medicines, CVD medicines, fat quality, fiber
HbA1c (mmol/l))	Diabetes medicines, fiber
Waist circumference (cm)	Gender
Systolic blood pressure mmHg	Education
Diastolic blood pressure mmHg	Gender, fruits and veg intake
BMI kg/m^2	-
