# Supplementary material for: Reallocating bouted sedentary time to non-bouted sedentary time, light activity and moderate-vigorous physical activity in adults with prediabetes and type 2 diabetes
Source: PLoS One. 2017 Jul 28;12(7):e0181053. doi: 10.1371/journal.pone.0181053 (PMC5533318; doi:10.1371/journal.pone.0181053)
Supplement: S2 Table — Bold indicates significant results (p<0.05) Activity intensity tresholds are <100 counts/min for sedentary (SB), 100 to 1951 counts/min for LPA and ≥1952 for MVPA. a Sedentary time was divided in 60 minutes or longer bouts (SB60) and non-bouted sedentary time (Non-bouted SB60). b Sedentary time was divided in 40 minutes or longer bouts (SB40) and non-bouted sedentary time (Non-bouted SB40). c Sedentary time was divided in 20 minutes or longer bouts (SB20) and non-bouted sedentary time (Non-bouted SB). (PDF) [file pone.0181053.s002.pdf]

	Regression coefficients (95 % CI)			Relative rate (95 % CI)				
	**Waist circumference** (cm)	**BMI** (kg/m^2)	**HDL cholesterol** (mmol/l)	**Diastolic blood pressure** (mmHg)	**Systolic blood pressure** (mmHg)	**HbA1c** (mmol/mol)	**Fasting plasma Glucose** (mmol/l)	**Triglycerides** (mmol/l)
Total Sedentary	**1.50 (0.54, 2.47)**	**0.42 (0.05, 0.79)**	**-0.04 (-0.07, 0.00)**	**1.01 (1.00, 1.02)**	1.00 (0.99, 1.01)	1.00 (0.99, 1.02)	**0.98 (0.96, 1.00)**	1.01 (0.97, 1.05)
LPA	**-1.26 (-2.37, -0.16)**	-0.32 (-0.74, 0.10)	0.02 (-0.01, 0.06)	**0.99 (0.98, 1.00)**	1.00 (0.99, 1.01)	1.00 (0.98, 1.01)	1.00 (0.99, 1.02)	1.00 (0.96, 1.04)
MVPA	**-4.33 (-7.14, -1.51)**	**-1.44 (-2.51, -0.36)**	**0.14 (0.46, 0.23)**	0.98 (0.96, 1.01)	1.11 (1.02, 1.04)	1.01 (0.96, 1.06)	1.01 (0.99, 1.03)	0.92 (0.82, 1.04)
SB60[a]	**1.14 (0.26, 2.03)**	0.32 (-0.02, 0.67)	-0.00 (-0.03, 0.03)	1.01 (1.00,1.02)	1.00 (0.99, 1.01)	1.01 (1.00, 1.03)	1.00 (0.99, 1.02)	0.99 (0.96, 1.03)
Non-bouted SB60	0.16 (-0.97, 1.29)	0.31 (-0.03, 0.64)	**-0.04 (-0.08, -0.01)**	1.01 (1.00, 1.02)	1,01 (1.00, 1.02)	0.99 (0.97, 1.00)	**0.98 (0.96, 1.00)**	1.02 (0.98, 1.07)
SB40[b]	**1.14 (0.42, 1.86)**	**0.34 (0.07, 0.62)**	0.01 (-0.04, 0.01)	1.01 (1.00,1.01)	1.00 (0.99, 1.01)	1.01 (1.00, 1.02)	1.00 (0.99, 1.01)	0.99 (0.96, 1.02)
Non-bouted SB40	-0.79 (-1.99, 0.41)	-0.29 (-0.74, 0.17)	-0.02 (-0.55, 0.02)	1.00 (0.99, 1.01)	1.00 (0.99, 1.01)	0.98 (0.96, 1.00)	0.99 (0.97, 1.01)	1.03 (0.99, 1.08)
SB20[c]	**1.02 (0.37, 1.67)**	**0.30 (0.06, 0.55)**	-0.02 (-0.04, 0.01)	**1.01 (1.00, 1.01)**	1.00 (1.00, 1.01)	1.00 (0.99, 1.01)	0.99 (0.98, 1.01)	1.00 (0.97, 1.03)
Non-bouted SB20	**-1.67 (-3.14, -0.20)**	**-0.56 (-1.11, -0.01)**	0.01 (-0.43, 0.06)	0.99 (0.98, 1.00)	1.00 (0.99, 1.01)	0.98 (0.96, 1.01)	1.00 (0.98, 1.03)	1.02 (0.97, 1.08)
