# Supplementary material for: Reallocating bouted sedentary time to non-bouted sedentary time, light activity and moderate-vigorous physical activity in adults with prediabetes and type 2 diabetes
Source: PLoS One. 2017 Jul 28;12(7):e0181053. doi: 10.1371/journal.pone.0181053 (PMC5533318; doi:10.1371/journal.pone.0181053)
Supplement: S3 Table — Bold indicates significant results (p<0.05) Activity intensity tresholds are <100 counts/min for sedentary (SB), 100 to 1951 counts/min for light intensity physical activity (LPA) and ≥1952 for MVPA. a Sedentary time was divided in 60 minutes or longer bouts (SB60) and non-bouted sedentary time (Non-bouted SB60). b Sedentary time was divided in 40 minutes or longer bouts (SB40) and non-bouted sedentary time (Non-bouted SB40). c Sedentary time was divided in 20 minutes or longer bouts (SB20) and non-bouted sedentary time (Non-bouted SB20). (PDF) [file pone.0181053.s003.pdf]

	Regression coefficients (95 % CI)			Relative rate (95 % CI)	
	**Waist circumference** (cm)	**BMI** (kg/m^2)	**HDL cholesterol** (mmol/l)	**HbA1c** (mmol/mol)	**Fasting plasma glucose** (mmol/l)
**Total SB**					
Total sedentary	0.55 (-0.34, 1.45)	0.05 (-0.30, 0.39)	-0.01 (-0.04, 0.02)	1.01 (0.99, 1.03)	1.00 (0.98, 1.02)
LPA	-0.47 (-1.64, 0.70)	0.00 (-0.04, 0.04)	0.00 (-0.04, 0.04)	1.01 (1.00, 1.03)	0.99 (0.97, 1.00)
MVPA	**-3.35 (-6.12, -0.57)**	**0.12 (0.03, 0.21)**	**0.12 (0.03, 0.21)**	1.02 (0.98, 1.07)	1.02 (0.97, 1.07)
**SB in 60 min bouts[a]**					
SB60	0.94 (-0.22, 2.10)	0.16 (-0.29, 0.61)	0.00 (-0.04, 0.04)	**1.02 (1.00, 1.04)**	1.00 (0.98, 1.02)
Non-bouted SB60	0.32 (-0.67, 1.31)	-0.02 (-0.40, 0.36)	-0.02 (-0.06, 0.01)	-0.02 (-0.06, 0.01)	**0.98 (0.97, 1.00)**
LPA	-0.21 (-1.47, 1.05)	-0.12 (-0.6, 0.37)	0.01 (-0.03, 0.05)	1.01 (0.99, 1.03)	1.01 (0.98, 1.03)
MVPA	**-3.36 (-6.14, -0.59)**	**-1.31 (-2.38, -0.24)**	**0.12 (0.03, 0.21)**	1.02 (0.97, 1.07)	1.02 (0.96, 1.07)
**SB in 40 min bouts[b]**					
SB40	0.99 (-0.04, 2.03)	0.22 (-0.18, 0.62)	-0.01 (-0.04, 0.03)	**1.02 (1.00, 1.04)**	0.99 (0.98, 1.01)
Non-bouted SB40	0.01 (-1.09, 1.11)	-0.16 (-0.58, 0.27)	-0.02 (-0.06, 0.02)	1.00 (0.98, 1.02)	**0.98 (0.96, 1.00)**
LPA	0.14 (-1.23, 1.50)	0.04 (-0.49, 0.56)	0.01 (-0.04, 0.05)	1.02 (1.00, 1.04)	1.01 (0.98, 1.03)
MVPA	**-3.39 (-6.15, -0.63)**	**-1.32 (-2.38, -0.26)**	0.12 (0.03, 0.21)	1.02 (0.97, 1.07)	1.02 (0.96, 1.07)
**SB in 20 min bouts[c]**					
SB20	0.72 (-0.21, 1.65)	0.13 (-0.23, 0.48)	-0.01 (-0.04, 0.02)	1.01 (1.00, 1.03)	0.99 (0.97, 1.01)
Non-bouted SB20	-0.45 (-2.25, 1.35)	-0.42 (-1.11, 0.27)	-0.03 (-0.09, 0.03)	0.98 (0.96, 1.01)	0.98 (0.95, 1.01)
LPA	0.25 (-1.36, 1.87)	0.15 (-0.47, 0.77)	0.01 (-0.04, 0.07)	**1.03 (1.00, 1.05)**	1.01 (0.98, 1.04)
MVPA	**-3.28 (-6.05, -0.51)**	**-1.27 (-2.34, -0.21)**	**0.12 (0.03, 0.21)**	1.02 (0.98, 1 .07)	**1.02 (0.97, 1.07)**
