# Supplementary material for: Reallocating bouted sedentary time to non-bouted sedentary time, light activity and moderate-vigorous physical activity in adults with prediabetes and type 2 diabetes
Source: PLoS One. 2017 Jul 28;12(7):e0181053. doi: 10.1371/journal.pone.0181053 (PMC5533318; doi:10.1371/journal.pone.0181053)
Supplement: S4 Table — Bold indicates significant results (p<0.05). Activity intensity thresholds are <100 counts/min for sedentary (SB), 100 to 1951 counts/min for light intensity physical activity and ≥1952 for MVPA. Total time in each intensity was divided by a constant of 30, so that one unit of increase corresponds to 30 min exchange between one activity to another. Total wear time was then entered together with covariates and all activities but one, simulating reallocation of 30 min in one activity by 30 min in another activity. By dropping each activity one by one the reallocating of time from different activities is modelled. a Sedentary time was divided in 60 minutes or longer bouts (SB60) and other sedentary time (Non-bouted SB60). b Sedentary time was divided in 40 minutes or longer bouts (SB40) and other sedentary time (Non-bouted SB40). c Sedentary time was divided in 20 minutes or longer bouts (SB20) and other sedentary time (Non-bouted SB20). (PDF) [file pone.0181053.s004.pdf]

	Regression coefficients (95 % CI)		
	**Waist circumference** (cm)	**BMI** (kg/m^2)	**HDL cholesterol** (mmol/l)
**TotalSB**			
Total sedentary to LPA	-1.02 (-2.11, 0.07)	-0.24 (-0.66, 0.18)	0.01 (-0.02, 0.05)
Total Sedentary to MVPA	**-3.90 (-6.72, -1.07)**	**-1.34 (-2.43, -0.26)**	**0.13 (0.04, 0.23)**
LPA to MVPA	-2.86 (-6.05, 0.34)	-1.10 (-2.32, 0.13)	**0.12 (0.01, 0.22)**
**SB in 60 min bouts[a]**			
SB60 bouts to non-bouted SB60	-0.62 (-1.78, 0.53)	-0.18 (-0.62, 0.27)	-0.03 (-0.07, 0.01)
SB60 bouts to LPA	**-1.15 (-2.27, -0.03)**	-0.27 (-0.70, 0.15)	0.01 (-0.03, 0.05)
SB60 bouts to MVPA	**-4.30 (-7.23, -1.38)**	**-1.46 (-2.60, -0.33)**	**0.11 (0.02, 0.21)**
Non-bouted SB60 to LPA	-0.53 (-1.95, 0.89)	-0.10 (-0.65, 0.45)	0.04 (-0.01, 0.08)
Non-bouted SB60 to MVPA	**-3.68, -6.54, -0.83)**	**-1.29 (-2.39, -0.19)**	**0.14, (0.05, 0.23)**
LPA to MVPA	-3.15 (-6.38, 0.08)	-1.19 (-2.43, 0.05)	0.11 (-0.00, 0,21)
**SB in 40 min bouts[b]**			
SB40 bouts to Non-bouted SB40	-0.97 (-2.15, 0.21)	-0.37 (-0.83, 0.08)	-0.01 (-0.05, 0.03)
SB40 bouts to LPA	-0.86 (-1.96, 0.25)	-0.18 (-0.60, 0.25)	0.02 (-0.02, 0.05)
SB 40 bouts to MVPA	**-4.38 (-7.25, -1.51)**	**-1.53 (-2.64, -0.43)**	**0.13 (0.03, 0.22)**
Non-bouted SB40 to LPA	0.14 (-1.62, 1.89)	-0.18 (-0.60, 0.25)	0.03 (-0.03, 0.08)
Non-bouted SB40 to MVPA	**-3.39 (-6.26, -0.52)**	**-1.15 (-2.26, -0.05)**	**0.14 (0.04, 0.23)**
LPA to MVPA	**-3.51 (-6.77, -0.24)**	**-1.35 (-2.60, -0.10)**	**0.11 (0.00, 0.22)**
**SB in 20 min bouts[c]**			
SB20 bouts to Non-bouted SB20	-1.17 (-2.99, 0.66)	-0.55 (-1.25, 0.15)	-0.02 (-0.08, 0.05)
SB20 bouts to LPA	-0.47 (-1.86, 0.92)	0.03 (-0.510, 0.60)	0.02 (-0.02, 0.07)
SB20 bouts to MVPA	**-3.4 (-6.82, -1.17)**	**-1.40 (-2.48, -0.32)**	**0.13 (0.04, 0.22)**
Non-bouted SB20 to LPA	0.75 (-2.15, 3.65)	0.59 (-0.53, 1.70)	0.04 (-0.06, 0.14)
Non-bouted SB20 to MVPA	-2.79 (-6.08, 0.49)	-0.84 (-2.09, 0.41)	**0.15 (0.04, 0.26)**
LPA to MVPA	**-3.51 (-6.85, -0.16)**	**-1.42 (-2.70, -0.13)**	**0.12 (0.03, 0.21)**
